# The impact of entrepreneurship research on other academic fields

**DOI:** 10.1007/s11187-023-00781-3

**Published:** 2023-05-22

**Authors:** A. Roy Thurik, David B. Audretsch, Jörn H. Block, Andrew Burke, Martin A. Carree, Marcus Dejardin, Cornelius A. Rietveld, Mark Sanders, Ute Stephan, Johan Wiklund

**Affiliations:** 1 Montpellier Business School, Montpellier Business School and LabEx Entreprendre of the Université de Montpellier, Montpellier, France; 2 Erasmus University Rotterdam, Rotterdam, the Netherlands; 3 Indiana University, Bloomington, IN USA; 4 Universität Trier, Trier, Germany; 5 Centre for Family Entrepreneurship and Ownership, Jönköping International Business School, Jönköping, Sweden; 6 Trinity College, Dublin, Ireland; 7 Universiteit Maastricht, Maastricht, the Netherlands; 8 Université de Namur, Namur, Belgium; 9 UCLouvain, Louvain-la-Neuve, Brussels, Belgium; 10 Erasmus University Rotterdam, Rotterdam, the Netherlands; 11 Universiteit Maastricht, Maastricht, the Netherlands; 12 King’s College London, London, UK; 13 Syracuse University, Syracuse, NY USA

**Keywords:** Entrepreneurship, Scientific impact, Academic fields, B00, L26, M13, O30

## Abstract

The remarkable ascent of entrepreneurship witnessed as a scientific field over the last 4 decades has been made possible by entrepreneurship’s ability to absorb theories, paradigms, and methods from other fields such as economics, psychology, sociology, geography, and even biology. The respectability of entrepreneurship as an academic discipline is now evidenced by many other fields starting to borrow from the entrepreneurship view. In the present paper, seven examples are given from this “pay back” development. These examples were first presented during a seminar at the Erasmus Entrepreneurship Event called *what has the entrepreneurship view to offer to other academic fields?* This article elaborates on the core ideas of these presentations and focuses on the overarching question of how entrepreneurship research impacts the development of other academic fields. We found that entrepreneurship research questions the core assumptions of other academic fields and provides new insights into the antecedents, mechanisms, and consequences of their respective core phenomena. Moreover, entrepreneurship research helps to legitimize other academic fields both practically and academically.

## Abstract (plain language summary)

Entrepreneurship research questions the core assumptions of other academic fields and legitimizes them both practically and academically. Since the 1980s, entrepreneurship research has seen tremendous growth and development, establishing itself as an academic field. Entrepreneurship is also taught extensively in leading business schools around the world. Indeed, few business schools do not address entrepreneurship in their curriculum. This represents a sea change: although entrepreneurs and new ventures had a remarkable impact on society, academia barely noticed it in the 1980s. Simply put: economics and business students rarely, if ever, encountered any mention of entrepreneurship during their studies. While entrepreneurship research has now developed its own methodological toolbox, it has extensively borrowed perspectives, theories, and methods from other fields. In the 2020s, we now find that entrepreneurship scholars are sharing its toolbox with other academic fields, questioning the core assumptions of other academic fields and providing new insights into the antecedents, mechanisms, and consequences of their respective core phenomena. Moreover, entrepreneurship research helps to legitimize other academic fields both practically and academically. Hence, entrepreneurship research now plays not just an important role in entrepreneurship education, practice, and policy but also throughout many other research fields.

## Introduction

Entrepreneurship research has seen tremendous growth and development over the last 4 decades and concomitant recognition as an academic field. The leading entrepreneurship journals receive ever more submissions from ever more countries, and their citation impact factors are on a steady rise. According to the 2021 impact factors, nine entrepreneurship journals are among the top hundred business and management journals included in the Social Sciences Citation Index (SSCI).^1^ Entrepreneurship is taught extensively in leading business schools throughout the world, and business schools without entrepreneurship courses in the curriculum simply do not exist. In summary, there is little doubt that entrepreneurship as a field has grown strongly and is now fully established.

Four decades ago, the situation was very different. Entrepreneurs and new ventures had a remarkable impact on society but were barely noticed in academia. It was unusual for both economics and business students to encounter any mention of entrepreneurship in their degree programs. On the whole, economics was embedded in a comparative static framework, where firms were assumed to maximize profits and where industry, technology, and consumer preferences were exogenously defined and unchangeable. There was no role for entrepreneurship in economic theory and little interest in entrepreneurship as a phenomenon.

The academic landscape started to change in the 1980s. Among the leaders pioneering this underexplored area were scholars who contributed to the establishment of the *Small Business Economics Journal* (SBEJ). Their new perspective did not take the existence of firms for granted or assume an infinite supply of capable entrepreneurs. Instead, they viewed the market entry of start-ups as essential to economies, albeit these startups were constrained in both number and capability due to limitations in their human and financial capital. Among other things, this new school of thought showed that economic performance depended on the ability for any industry to have a sufficient number of capable start-ups. For the first time, economic analysis was relevant for economic policy prioritizing entrepreneurship.

Beyond market entry, these scholars offered a more dynamic perspective of post-entry industry performance. In addressing the evolution of industries, new research questions were addressed well beyond the initial visions. For example, strategic entrepreneurship and entrepreneurial finance have evolved as subfields with their own academic journals.

Early entrepreneurship research extensively borrowed perspectives, theories, and methods from other fields but has now developed its own toolbox. The next step in the maturation of entrepreneurship research would be to start giving back to these other fields. Have we reached that point yet? Can we now, several years after the birth of entrepreneurship research, also observe knowledge flows in the opposite direction? Or, in a more provocative way, does anybody outside the field of entrepreneurship really care about entrepreneurship research? Answering these questions is important because it helps to further evaluate the state of entrepreneurship as an academic field and establish its academic legitimacy.

To answer these questions, a seminar called “What has the entrepreneurship view to offer to other academic fields?” was organized at the Erasmus School of Economics for a group of scholars to reflect upon how entrepreneurship research relates to other academic fields. The following three specific questions were at the core of the discussion: (1) what did the field look like before contemporary entrepreneurship research? (2) How has contemporary entrepreneurship research influenced/impacted the field? (3) How will this influence evolve in the future/in the next few years?

The specific fields represented are (clinical) psychology, (occupational) health, genetic epidemiology, culture, industrial organization, macro-economics, and public policy. We do not claim that the fields chosen are fully representative of the entire spectrum of fields upon which entrepreneurship has an impact.^2^ What matters is that the fields are very different in their theories and methods as well as in their research traditions and research culture. Taken together, they clearly illustrate the wide range of academic fields with which entrepreneurship as a field interacts.

The next seven subsections describe the respective view on the impact of entrepreneurship research on the different fields.^3^ After that, we provide structure and categorization to the insights and provide an overview of the impact that entrepreneurship research has had on these as well as other fields. The final section concludes and provides an outlook on the paths and scenarios of how entrepreneurship and its neighboring (and not so neighboring) fields and disciplines can co-evolve. While the impact of entrepreneurship research varies considerably across specific academic fields, what emerges from this article is an undeniable impact. Research has not only transformed what we know and understand in the scholarly field of entrepreneurship but thinking across a broad spectrum of other fields has also developed considerably.

## Entrepreneurship and its relationship with other academic fields

### Entrepreneurship and (clinical) psychology

Borrowing without giving back is not truly borrowing. It is stealing. We often say that entrepreneurship borrows from other areas of research. However, we rarely clarify what entrepreneurship brings back to these other fields or disciplines. Perhaps we sometimes steal? Clinical psychology is an area from which entrepreneurship has started borrowing increasingly over the past decade (see, for example, Gish et al. (2022)). However, it is also an area where it is evident that entrepreneurship is bringing much back to clinical psychology. Specifically, we will discuss how the development of research within entrepreneurship regarding the associations between entrepreneurship and the clinical condition of attention deficit hyperactivity disorder (ADHD) contributes to the evolution of clinical psychology.

Clinical psychology represents normal science as defined by Thomas Kuhn. This means that within the field, there is great agreement on what kind of research belongs within the field and what does not; which methods are appropriate, and which are not; and which theories should be applied and which should not. For a recent example pertaining to ADHD, see, for example, the review by Song et al. (2021). The bulk of research follows a “coherentist” approach, where coherence among theory, measurement, and statistical analysis takes priority over new observational knowledge (Tal, 2013). This is fruitful, as the validity of findings is contingent on whether constructs hold up (cohere) with established theory and facilitate the accumulation of knowledge. The downside is that new phenomena receive scant attention and that observational anomalies tend to be discounted. Entrepreneurship research represents its antipode. There are few established definitions, methods, and theories. An attitude of “anything goes” characterizes the field (Wiklund et al., 2019). For many clinical psychologists, the field appears confusing, sloppy, and not as real science.

Given these fundamental differences between the fields, it is no surprise that studies of potential associations between ADHD and entrepreneurship have been observed by entrepreneurship scholars and not clinical psychologists. Of the 62 peer-reviewed journal articles to date on the potential association between ADHD and entrepreneurship, 61 appear in the entrepreneurship and management journals and not even one in a clinical psychology publication. Across a variety of samples, utilizing various methods and measures, a pattern of results has started to emerge that calls into question some of the fundamental assumptions made by clinical psychologists regarding ADHD. Findings from this stream of research suggest that people with ADHD diagnoses or ADHD symptoms are attracted to entrepreneurship as an occupation (Verheul et al., 2015), that they are likely to actually engage in entrepreneurial endeavors (Verheul et al., 2016) and that they may even perform well in entrepreneurship (Yu et al., 2021). These research questions lie outside the mainstream of what clinical psychology concerns itself with; the findings represent an anomaly to fundamental theory and are often derived using methods that are not standard within the field. Perhaps most interesting is the finding that a clinical condition that is associated with many challenges may actually be associated with certain strengths within entrepreneurial endeavors. We believe these are findings that have extensive practical and theoretical implications.

It is within these areas of research, outside of the mainstream of normal science that entrepreneurship scholarship truly shines. Scholars have been willing to ask novel and relevant research questions, to use whatever data they could find, and to analyze them using a variety of methods. Unless some scholars are willing to accept such tradeoffs between relevance and rigor, new important phenomena will remain unexplored, and scholars risk becoming increasingly locked into ivory towers. We have provided ADHD as an example where entrepreneurship scholarship informs clinical psychology. Similar arguments can be made regarding the relationship between dyslexia and entrepreneurship, where Julie Logan pioneered research showing positive relationships that clinical psychologists had hitherto overlooked (Logan, 2009).

Therefore, what comes next? *First*, there are indications that clinical psychologists indeed are becoming more interested in researching entrepreneurship and ADHD. This includes citation to the work conducted by entrepreneurship scholars, entrepreneurship scholars delivering keynotes at clinical psychology conferences, and informal discussions about researching the topic during these conferences. We also know that papers on the topic are submitted to clinical psychology journals, even though they have not yet been published there. We should keep in mind that this is a rapidly evolving field of research. Our review located 5 articles published between 1993 and 2015 and 57 articles published between 2016 and 2022. As a normal science, clinical psychology is slow to change. *Second* and more important, we believe this area of research on the links between entrepreneurship and ADHD will take long strides forwards when scholars in entrepreneurship and clinical psychology come together, asking the novel and relevant questions that entrepreneurship scholars are good at and applying the methodological rigor of clinical psychology. Thus, this research has the potential to make it into mainstream clinical psychology. Such a development would be an excellent example of how entrepreneurship research can advance clinical psychology research.

### Entrepreneurship and (occupational) health

Occupational health and related fields (occupational medicine, occupational health psychology) traditionally study work-related risk factors of disease. With the emergence of a broader understanding of health as “… a state of complete physical, mental and social well-being and not merely the absence of disease or infirmity” (World Health Organization, 1948), interest in mental well-being—positive experiences and functioning—and its drivers grew (Antonovsky, 1979; Warr, 1987). Today, largely parallel lines of research focus on how work stress produces health impairments (Ganster and Rosen, 2013) or on the motivational effects of work and well-being (Judge et al., 2017).

The study of health and well-being by entrepreneurship researchers has accelerated over the past decade (for reviews see Stephan, 2018; Stephan et al., in press a; Torrès and Thurik, 2019; Wiklund et al., 2019). More recently, the COVID-19 pandemic drew further attention to health and well-being, including that of entrepreneurs (Batjargal et al., in press; Stephan et al., in press b; Torrès et al., 2021). For occupational health and related fields, studying entrepreneurship (1) helps to integrate the research lines on health impairments (ill-being) and well-being, (2) uniquely offers insights into the future of work, (3) provides opportunities to showcase the relevance of health/well-being for economic and social outcomes, and (4) reinforces the need to expand occupational health and related fields to the study of (strategic) leadership.

Entrepreneurship is an “extreme” work setting that is often simultaneously intensely stressful and fulfilling (Baron, 2010; Stephan, 2018; Wiklund et al., 2019)—often described as a “rollercoaster” by entrepreneurs. Compared to employees who are studied in occupational health, entrepreneurs’ work involves longer working hours (the longest of any occupation), higher workload, more time pressure, and often more loneliness and financial worries. Entrepreneurs’ work also entails more uncertainty and frequently changing demands (e.g., slumps in market demand). In addition to stressors, entrepreneurs’ work is also more extreme in regard to well-being resources. Because entrepreneurship combines ownership and control, entrepreneurs have a degree of independence and autonomy that is difficult to find in other occupations. They can decide with whom, on what, when, and how to work. This autonomy allows entrepreneurs to shape work in line with their values, skills and knowledge—much more so than is possible for typical employees—which makes work more meaningful for entrepreneurs. Autonomy and meaning enhance entrepreneurs’ sense of personal responsibility, which leads them to further invest in their work, often neglecting their private life, family and friends and granting themselves little respite from work.

Because it is an extreme work setting, entrepreneurship can uniquely enhance both ill-being and well-being, leading to a so-called well-being trade-off (Stephan et al., in press a) that is not yet recognized in occupational health research. The high levels of stressors that entrepreneurship entails trigger health impairment processes accompanied by feelings of distress, enhanced levels of stress biomarkers and, with a time delay, ultimately stress-related mental and physical disease (Rauch et al., 2018). At the same time, the high levels of well-being resources simultaneously trigger motivational processes and “happiness.” Thus, studying entrepreneurship expands occupational health research that has often ignored the potential negative effects of well-being resources such as autonomy and meaning (for an exception: Warr, 1994) and integrates the study of well-being and ill-being because without considering both, detrimental health effects go unnoticed (Grant et al., 2007).

The features of entrepreneurial work make entrepreneurship an excellent *laboratory in which to understand the health/well-being effects of the future of work*. The future of work is marked by more uncertainty, more variable and dynamically changing work demands, blurring of work-life boundaries, and personal responsibility, i.e., all hallmarks of entrepreneurs’ work. Because research on entrepreneurs’ health/well-being draws attention to new stressors and resources beyond those typically considered in occupational health research, it is uniquely positioned to offer insights into both the “bright” and the “dark” side of the future of work, into both thriving and fulfillment but also strain and precariousness. Moreover, entrepreneurship calls attention to well-being trade-offs and thus that thriving can be intimately linked to strain. At the same time, entrepreneurs are at the frontline of creating the future of work. For instance, start-ups instigated fully remote work and flexible hours to enhance employee wellbeing before the COVID-19 pandemic. In another example, entrepreneurial firms use strength-based organizational design to create inclusive work for those living with disabilities. Of course, entrepreneurship is itself part of the future of work as more individuals become entrepreneurs.

Entrepreneurship is also *an exemplary context in which to document the relevance of health/well-being for economic and social outcomes.* For occupational health research interested in making a case that health/well-being “matters,” entrepreneurship provides a fruitful context. Because the organization that an entrepreneur creates is an expression and extension of herself or himself, entrepreneurs’ health/well-being matters more directly for organizational productivity, growth, and survival than the health/well-being of individual employees matters for the same organizational outcomes. Similarly, the social consequences of health/well-being may be particularly salient in an entrepreneurship context, for instance, the way entrepreneurs’ own health/well-being impacts other stakeholders from employees, customers, suppliers to investors (Bort et al., 2020) or how it may lead to (un)ethical business practices and even positive social change by fostering positive business climates and tolerance (Tobias et al., 2013). Thus, studying entrepreneurs’ health/well-being substantially widens the scope of social outcomes beyond those typically considered in occupational health (such as spillover effects on life partners, family, and hobbies).

Finally, studying entrepreneurs’ health/well-being also offers *new insights into research on strategic leadership*, such as how the health/well-being of C-level executives may shape strategic decision-making and firm performance. Strategic leaders have been absent from occupational health research (although there is growing interest in how (middle-management) leadership intersects with health/wellbeing; Inceoglu et al. (2021)). Equally, strategy research has not yet considered the health/well-being of strategic leaders. Entrepreneurs and strategic leaders face a similar challenge—to stay productive despite the intense and persistent stresses of their work. Recent contributions point to the importance of self-care and stress recovery routines for such work settings, which present opportunities for new academic insights with practical relevance (Weinberger et al., 2018; Williamson et al., 2021).

In sum, entrepreneurship draws attention to neglected and understudied areas of occupational health research. Emerging research documents the potential for research at the intersection of entrepreneurship and health to advance new insights and to “future-proof” health research for an increasingly dynamic and uncertain world of work.

### Entrepreneurship and genetic epidemiology

Thousands of heritability studies analyzing a large variety of traits (i.e., observable characteristics) have led to the formulation of the “first law” of behavior genetics stating that all human behavioral traits are heritable (Turkheimer, 2000; Polderman et al., 2015). With entrepreneurship as another instance of a behavioral trait, it is not surprising that several studies have found evidence for its heritable nature (Nicolaou et al., 2008; Nicolaou et al., 2008; Nicolaou and Shane, 2010; Van der Loos et al., 2013; Zhang et al., 2009). Heritability estimates capture the proportion of observed differences in a trait among individuals from a certain population that is due to the genetic differences among these individuals (Visscher et al., 2008). The completion of the sequencing of the human genome some 20 years ago now (Venter et al., 2001) kick-started the large-scale collection of genetic data in medical cohort studies. With these genetic data, genetic epidemiologists started to search for the specific genes influencing heritable diseases and traits such as heart disease, diabetes, auto-immune diseases, and psychiatric disorders (Visscher et al., 2012). Motivated by the idea that matches and mismatches between genetic predispositions and career choices could impact morbidity and mortality (Koellinger et al., 2010; Van der Loos et al., 2010), an international consortium was set up to analyze the molecular genetic architecture of entrepreneurship using genome-wide association study (GWAS) meta-analysis (Van der Loos et al., 2011; Van der Loos et al., 2013).

Despite the heritable nature of entrepreneurship and the fact that entrepreneurship tends to run in families (Andersson and Hammarstedt, 2010), no single robust and replicable association between a genetic variant and entrepreneurship has been identified by this “Gentrepreneur consortium”. Many individuals attempt to set up a business at least once in their life, making it not surprising that a large-scale GWAS meta-analysis based on mostly cross-sectional data about someone’s current self-employment status from various countries did not result in statistically significant estimates of relationships between genetic variants and entrepreneurship (Van der Loos et al., 2013). However, to capitalize on lessons learned, with others, the Gentrepreneur consortium members also established the Social Science Genetic Association Consortium (SSGAC) with the aim of analyzing the genetic architecture of behavioral outcomes more comprehensively. For the first time in history, the SSGAC identified genetic variants that were robustly associated with a behavioral outcome (Rietveld et al., 2013). Although only three genetic variants with tiny effects on educational attainment were found, this study is commonly appreciated as a “game-changer” (Bliss, 2018): This study showcased the possibility of finding replicable genetic associations with behavioral outcomes if sufficiently large analysis samples are gathered and analyzed.

The ongoing publication of successful GWASs on behavioral outcomes raised the question of whether the estimated genetic effects should be attributed to biological factors or to environmental factors through which these genes operate (Bliss, 2018). Answering this question is not straightforward. For instance, although the genetic variants associated with educational attainment relate to biological pathways involved in neural development (Okbay et al., 2016), their effects are dependent upon the presence of a schooling system in which differences in endowments tend to lead to particular educational trajectories. Similarly, genetic effects on entrepreneurship are not expressed in a communist country where private enterprising is not allowed. Thus, the effects of “nature” and “nurture” are not additive and separable but intrinsically intertwined and nonlinear (Biroli et al., 2022).

This is where entrepreneurship research, or economic research more generally, can contribute to genetic epidemiology and social science genetics. A growing stream of literature is using gene-by-environment (G×E) analysis to investigate the dependency of genetic effects on conditions in the environment (Pereira et al., 2022). For example, G×E studies can be used to identify (institutional) environments that compensate for genetic disadvantages (Barcellos et al., 2018) or that amplify the production of human capital (Muslimova et al., 2021). Economic tools are of particular relevance to better design G×E studies for behavioral outcomes (Biroli et al., 2022). The reason is that, empirically, both genetic endowments and environmental factors are likely to be endogenous when modeling a particular behavioral outcome (Keller, 2014). Economists have developed a large toolbox (containing, e.g., regression discontinuity designs and instrumental variable regression) to address endogeneity, and they have a long tradition of exploiting exogenous variation in environmental exposures to estimate causal effects. The entrepreneurship literature is replete with studies analyzing the effects of exogenous changes in the environment on entrepreneurship phenomena (Block et al., 2012; Block et al., 2013; Hoogerheide et al., 2012). Therefore, economists and entrepreneurship researchers are well positioned to improve the understanding of the complex interplay between nature and nurture in shaping behavioral outcomes.

Eventually, well-designed G×E studies can also reveal how matches and mismatches between genetic predispositions and entrepreneurship could impact stress and health. Then, we are back at where the “quest for the entrepreneurial gene” (Van der Loos et al., 2011) started, but no longer to analyze how genetic predisposition for entrepreneurship may moderate the impact of work conditions on health and morbidity but to analyze how exogenous changes in a broadly defined entrepreneurship-relevant environment may heterogeneously impact genetically different individuals. For this purpose, from a genetic perspective, one can draw on summary indices of genetic variants (so-called polygenic scores or polygenic indices) that are increasingly available in well-known, publicly available datasets (Becker et al., 2021). These polygenic scores are constructed for particular traits, such as educational attainment, subjective well-being or attention deficit hyperactivity disorder (ADHD), and can be used to reveal which combination of genetic endowments and environmental circumstances impact entrepreneurship and person-job fit (Rietveld et al., 2021; Patel et al., 2021; Patel et al., 2021).

### Entrepreneurship and culture

Culture is not a discipline or field but part of a context that contributes to shaping entrepreneurship and determining its consequences and impacts (Welter, 2011; Baker and Welter, 2020). Consequently, if we want to know more about entrepreneurship and be able to appreciate it in all its aspects and variety, culture is a major point of attention, and it is essential that research in entrepreneurship takes culture into account.

Many academic disciplines have studied culture, and many definitions of culture can be found in social sciences. The historical but seminal definition of Tylor (1871, p. 1) and taken up by Lévi-Strauss (1958) in anthropology describes culture as “that complex whole which includes knowledge, beliefs, arts, morals, law, customs, and any other capabilities and habits acquired by man as a member of society.” In sociology, a discipline with a keen interest in culture, Steensland (2018) defined it as follows: culture “refers to the beliefs that people hold about reality, the norms that guide their behavior, the values that orient their moral commitments, or the symbols through which these beliefs, norms, and values are communicated.” In economics, culture is also an object of interest and has been for a long time. Without going too far, we cannot ignore the work of institutional economists who define culture as a set of informal rules and distinguish them from formal institutions (Alesina and Giuliano, 2015).

For Guiso et al. (2006, p. 23), culture is “those customary beliefs and values that ethnic, religious, and social groups transmit fairly unchanged from generation to generation.” This definition by economists contrasts sharply with that which can be found in the so-called “cultural studies,” “concerned with an exploration of culture, as constituted by the meanings and representations generated by human signifying practices, and the context in which they occur. (…) (With) a particular interest in the relations of power and the political consequences that are inherent in such cultural practices” (Barker, 2004, p. xix).

Apart from the cultural studies approach, culture appears to be a persistent contextual element. This element is not ignored by entrepreneurship research. Culture is generally identified here as an explanatory factor that can be linked to entrepreneurial phenomena by relying on competing or complementary theoretical approaches (see Thurik and Dejardin (2012) for a brief survey), in particular post-materialism (Inglehart, 2003), dissatisfaction (Brockhaus, 1980; Shapero and Sokol, 1982; Baum et al., 1993), aggregated psychological traits (Davidsson, 1995; Obschonka et al., 2015), and social legitimation (or moral approval) (Etzioni, 1987; Baumol, 1990).

Culture is a pervasive element in general conceptualizations linking entrepreneurship, its context and economic development. See, for example, the Global Entrepreneurship Monitor conceptual framework (GEM, 2022); foundations behind the Global Entrepreneurship Index, its regional variation (GEDI, 2019; Szerb et al. 2013) and the entrepreneurial ecosystem view (Stam and Spigel, 2018).

Empirical studies in entrepreneurship research linking culture to entrepreneurship have multiplied in recent years, especially studies relying on the aggregation of psychological traits and those addressing, among others, the question of entrepreneurial persistence characterizing some regions (Fritsch and Wyrwich, 2014; Stuetzer et al., 2016, Stuetzer et al., 2018; Audretsch et al., 2017; Fritsch et al., 2021; Obschonka et al., 2021).

However, as is often the case in research, new issues emerge as we move forwards. We can think of the links between culture and entrepreneurship in their persistent or change aspects (Voth, 2021). In particular, it would be welcome to investigate the mechanisms at work and what the conducive channels are and to progress in the measurement of these mechanisms. Moreover, alongside the aggregation of psychological traits approach, might we also get a little closer to a more holistic approach of culture, in particular by examining beliefs and values that are actively shared by individuals?

To go a little further, an appeal can be extended to the proponents of the research on culture. If culture, as an element of context, contributes to determining entrepreneurship (not only its intensity but also its kind and meaning), one should not rule out entrepreneurship as a potential explanatory factor in turn. Entrepreneurship may affect formal and informal institutions such as culture, as suggested by Feldman et al. (2005). Referring to the US Capitol region in the 1980-90s, these authors report how entrepreneurs appeared to be a critical factor in the formation of dynamic biotechnology and ICT clusters. It took 2 decades to achieve self-sustaining development with strong industry networks and a supportive local culture. Another example is provided by Thompson et al. (2018) regarding the emergence of entrepreneurial ecosystems. By carefully analyzing social impact initiatives in Seattle, they document how everyday interactions among a set of individuals can endogenously lead to building an entrepreneurial ecosystem and the sharing of beliefs and values supportive of the whole.

Entrepreneurs can be highly involved in creating new institutional and cultural combinations, which also opens new perspectives for understanding change (Lowe and Feldman, 2017). After all, what makes one region seem more entrepreneurial than another and a lasting success? It is undoubtedly the result of multiple intertwined factors, constituting a complex system which requires in-depth study, as Saxenian (1994) was able to propose, adopting a multidisciplinary point of view, in her now classic contribution. Beyond the emblematic Silicon Valley, Route 128, or the Tel Aviv region, there may be many more examples. Are we not often quoting culture as an explanatory hypothesis? The local culture would be more entrepreneurial, e.g., being more oriented towards a culture of entrepreneurial financing or towards entrepreneurial innovations. These conjectures lead to questioning what an entrepreneurial culture is, how it interacts with other aspects, and how to develop it (Beugelsdijk, 2010; Hayton and Cacciotti, 2013; Marti et al., 2013). We still know little about it.

To this batch of questions could also be added: “where is entrepreneurship in the examination of emerging cultural phenomena?” What fits best: considering entrepreneurship as an endogenous cultural factor or as an exogenous, disruptive one? Of course, everything relates to everything in social reality.

### Entrepreneurship and industrial organization

The focus of entrepreneurship research appears largely complementary to that of the field of industrial organization. Although both areas of research are concerned with the supply side of the economy, the attention of entrepreneurship scholars is usually on small entrants, whereas the study of industrial organization scholars is usually that of large incumbents. Even in the case when entry as a topic is presented in industrial organization textbooks, it deals with diversification, entry barriers and entry deterrence, mainly from the focus of incumbents. This emphasis on large market players can also be seen in typical measures of “structure” such as the C4 concentration index and the Herfindahl index, which more or less neglect small firms. In this section, some aspects of entrepreneurship research especially relevant to complementing and changing our view on industry structure, conduct and performance are presented. First, the differences in scientific tradition are elaborated upon. After that, there are three questions largely neglected by industrial organization scholars but prominent in entrepreneurship research: who starts firms? Where do industries come from? How do firms innovate and compete?

#### Differences in scientific tradition

In 1989, when SBEJ started and the economics of entrepreneurship was still in its infancy, the field of industrial organization was already mature. This can be seen in still state-of-the-art books, such as Tirole (1988), Schmalensee and Willig (1989) and Sutton (1991). Since the 1930s, industrial organization research has increased our understanding of the supply side of the economy, especially in regard to the structure of industries, interdependence of firms and their choice of actions, and welfare consequences. The study of industrial organization is directly connected to micro-economics, has a clear area of application in competition policy and is, therefore, an indispensable element of economics as an academic discipline. It is also very “mainstream” (neo-classical), as it assumes rational (profit maximizing) players and tends to analyze equilibrium outcomes. The interdependence of the market players is analyzed through game theory, making industrial organization a relatively mathematics-heavy research area.

Entrepreneurship was more or less defined away in mainstream economics (see, e.g., Baumol, 1968), and its role was reduced to that of “assuming risk” (Kihlstrom and Laffont, 1979), but there were two important exceptions, both reasoning “out of equilibrium” and therefore emphasizing a much more dynamic role. Schumpeter claimed the role of entrepreneurs to lie in choosing new combinations in production (innovation) and in disrupting the steady state, while Kirzner (1985) emphasized the alertness to and exploitation of profit opportunities that result from (continuously emerging) disequilibria, thus moving the economy towards equilibrium (Hébert and Link, 1989). The difference in emphasis on a dynamic interpretation of the market versus a more static one has important implications. Models in industrial organizations usually take the current market structure and a certain variable on which players compete (e.g., price or product quantity) and solve for the equilibrium outcome. Entrepreneurship research is more focused on new entrants with new products and novel ways of competing, changing the market place, and making game theoretic analysis a less obvious tool.

Whereas industrial organization is part of economics, entrepreneurship research is much more interdisciplinary. Various insights and contributions of other social sciences, such as psychology and sociology, influence the field. An example of how this can enrich our knowledge of the exit of firms is an article by Lahti et al. (2019) using brain imaging to show that entrepreneurs tend to bond to their firms so that when it would not be economically wise to continue, firms may remain in existence far beyond what models assuming rationality would predict.

#### Who starts firms?

In addition to being more focused on market dynamics, a prominent difference between industrial organization and entrepreneurship research is that the latter is very interested in who actually starts (or discontinues) firms. The “human aspect” of a firm is largely irrelevant to an industrial organization researcher who regards it as a market player acting rationally, as in maximizing profit. However, Wach et al. (2016) provide strong empirical evidence that entrepreneurs tend to have multiple goals exceeding financial ones. This may impact the over-reliance on the assumption of (hyper) rationality in game theoretic analyses using one-dimensional (profit) objectives for the players. A multidimensional individual-specific objective function not only renders the assumption of profit maximization problematic but that also holds for the assumption that in their strategic choices, firms consider profit maximization by the other parties. Stenholm and Renko (2016) emphasize another important aspect distinguishing successful from less successful entrepreneurs: flexibility. They find that passionate bricoleur (i.e., flexible) entrepreneurs outperform other entrepreneurs, as they can cope better with changing market circumstances (e.g., crises). The importance of flexibility over economies of scale and scope was already suggested as one of the key advantages for small firms in the early days of small business economics (e.g., Fiegenbaum and Karnani, 1991). However, some entrepreneurs may be more flexible because of their social network and skills. Lazear (2004) and Stuetzer et al. (2013) suggest that those who are more balanced in their skills are more likely to become entrepreneurs. Being balanced in skills may also help adjust the company when market circumstances alter. Baron (2006) connects opportunity recognition to the background of the entrepreneur. Entrepreneurs who were already working in the industry would, for example, be better at recognizing new opportunities due to changes in the market. Adomako et al. (2018) show that it is not only opportunity recognition on its own but also the connection with useful networks that makes firms excel. This finding shows that flexibility is likely to differ across market players. Again, this makes the outcome of highly stylized game theoretic analyses of company interaction less useful.=

#### Where do industries come from?

The origin of an industry would seem to be an obvious research question in the field of industrial organization. However, it has been more or less neglected in mainstream industrial organization research. In 1982, three important publications emphasized the evolution of the industry. Those were the highly stylized formal model by Jovanovic (1982), much in line with neo-classical theory; the book by Nelson and Winter (1982), setting the stage for a range of evolutionary studies; and the empirical analysis by Gort and Klepper (1982). Klepper was an especially influential scholar in the field of entrepreneurship research. He has contributed more to answering the question of where industries emerge from than any other scholar. He called his detailed method of investigation “nano-economics” (Klepper, 2011) and showed the importance of pioneers and spinoffs. His study of industries such as automobiles, tires, semi-conductors and garments in India has vastly increased our understanding of where industries originate. The importance of universities and science has obviously increased over time, and Buenstorf and Heinisch (2020) provide an example of how it impacted the start of the German laser industry. Karlsson and Nyström (2003) note that knowledge intensity is especially important in the early stages of the life cycle. The origin of industries is even more important because there is strong path dependency. Pioneering firms are the seedbed for entire industry clusters decades later. Industrial organizations tend to start taking notice of a cluster when it is also very mature, but the entrepreneurial first steps are critical in themselves.

#### How do firms innovate and compete?

R&D and patents are high on the agenda in industrial organizations with regard to measuring innovation. However, the way of innovating and the protection of its results are quite different for new and small firms—often lacking financial means—compared to their larger counterparts. Acs and Audretsch (1988) were among the first to recognize the difference between large and small firms in innovation. Many (small) firms innovate without reporting substantial R&D expenses. Leiponen and Byma (2009) discuss how small firms differ from large firms in regard to protecting their innovations (if they do so). Entrepreneurs may benefit from innovations by large firms, which is important in the knowledge spill-over theory by Acs et al. (2009). They are also likely to depend much more on external sources of knowledge. Carree et al. (2019) show how small firms may benefit more from a small number of cooperations when compared to large firms. In the era of open innovation, having a well-developed network of innovation partners is vital for new and small firms to introduce new products and production technologies. Industrial organization scholars have tended to confine their attention to research joint ventures (of two parties). The benefits of cooperation in R&D versus reduced competition after R&D produce interesting welfare considerations. However, this line of inquiry provides little insight into the dynamic networks firms in high-tech industries participate in.

Competition is at the heart of industrial organization research. The means of analyzing competition is very much conditioned by oligopoly models and game theoretical approaches. Depending upon how firms competitively interact, they may achieve low or high profits. A simple Bertrand oligopoly with homogeneous goods, for example, ends with zero profit irrespective of the number of market participants. This is not attractive to an entrant, and new firms may seek to avoid competition by introducing a new product that may more or less substitute for the current one. Gans et al. (2018) discuss various ways in which new firms may enter into existing markets. Such entry normally does not follow “the rules of the game.” Many new firms want to introduce a product that does not yet exist in the market, thereby reducing price competition (Kim and Mauborgne, 2004). The extent to which a product is unique often derives from how special the human and social capital of the entrepreneurs is. Competition is often not so much over current products but much more over ideas (Chatterjee and Rossi-Hansberg, 2012). Markets have become much more volatile and interdependent. Highly stylized game theoretic models—even how useful in our understanding of “basic” situations—tend to stand further and further away from this new reality. The way entrepreneurship research has been thinking about “competition,” in a more dynamic, Schumpeterian or Kirznerian way, seems much closer.

Entrepreneurship research enriches the field of industrial organization to actively go beyond it being reduced to “applied game theory.” The actual behavior by firms, their emergence and growth and the dynamics of industries are topics of high interest that benefit considerably from scientific contributions from the field of entrepreneurship. The (potential) added-value of entrepreneurship research to industrial organization and their fruitful interaction has not gone unnoticed of course, see for example a recent special issue in Review of Industrial Organization (November 2020). In the upcoming years the field of Industrial Organization, which has been so instrumental in the development of small business and entrepreneurship research, will itself in return profit from new insights.

### Entrepreneurship and macro-economics

#### Entrepreneurship is absent in macro-economics

Macro-economics has focused largely on questions of long-run economic growth and development in recent decades. For a long time, neoclassical mainstream economics had trouble explaining long-run economic growth. Diminishing returns to capital, combined with linear depreciation, implied that in the long run, the capital stock stabilizes, and perfect competition leaves no resources available for creating growth. Technical change and productivity growth were modeled but not explained. This unsatisfactory situation ended with the arrival of endogenous growth theory. In these models, the creation and introduction of new ideas, financed from temporary monopoly profits in monopolistic markets, creates the flow of innovations that sustains long-run equilibrium growth (Romer, 1990; Jones, 1995). This “leap” in macroeconomics was made possible because others developed models for imperfect competition (Barro and Sala-I-Martin, 2003). However, while the work of Schumpeter inspired some to present a model of creative destruction (Aghion and Howitt, 1998), endogenous growth theory still did not bring entrepreneurship to the center stage. Instead, macro-economists’ attention shifted to institutions (Acemoglu, 2009) and left entrepreneurship remain marginalized from mainstream thinking.

The reason is not that macro-economists are daft and dumb and were too stupid to see the obvious. In macro-economics, agents are defined by what they do and are only relevant to the extent that what they do has aggregate implications. Consumers are defined as agents that consume and save. Investors are agents that invest in physical or human capital, and producers hire capital and labor to produce output. The reason that entrepreneurship has contributed little to macro-economics to date is our joint inability (or unwillingness) to come up with a sensible, unambiguous, and most importantly, non-tautological definition of what it means to be an entrepreneur (Sanders, 2022). This implies that modeling entrepreneurial activity in a macro-economic model is impossible, and much of our empirical evidence could be set aside as trivial. Yes, growth and entrepreneurship (however, proxied) correlate at the national level (Wennekers and Thurik, 1999), but so do a host of other variables. These are all proximate causes of growth and, as such, not that interesting. The way a macro-economist now looks at entrepreneurs is that there is always an entrepreneur who will pick up a valuable idea. There is also a worker who will fill a vacancy and an investor who will fund a positive net present value investment. All we need is put in place an incentive for them to do so (a free-entry and zero profit condition), and it is productivity, ultimately caused by knowledge creation (and the institutions that promote or hinder it), that causes capital, labor, entrepreneurship and all other proximate causes to grow with it.

#### … but this can change …

Taking Schumpeter (1911; 1942) more seriously, however, entrepreneurship research has taught us that creating an innovation from an invention is far from trivial. Building an organization, refining the concept into a value proposition, and accessing and mobilizing resources to deliver that value to customers is a deeply uncertain, context-dependent, and potentially very costly affair. The people undertaking such activities are in short supply, and the success of those who try depends crucially on their traits, talents, environmental circumstances, and luck (Bosma et al., 2004; Coad and Story, 2021). There are two reasons why we may be hopeful that these insights will now also enter the world of macro-economics.

First, advances in computing power make it possible to design, run, and analyze models of increasing complexity that allow for more heterogeneity. Not unlike the advances in modeling imperfect competition, agent-based modeling (De Marchi and Page, 2014) now allows macro-economists to explore (relevant) heterogeneity among agents they previously were forced to consider homogenous and representative. This has already revolutionized the modeling of financial and labor markets. We know that entrepreneurs are not homogenous and cannot usefully be represented in representative agent models. Indeed, if entrepreneurship is reduced to a predictable process in the aggregate (as in all representative agent endogenous growth models), the essence of entrepreneurial venturing will be lost. However, to feed into this emerging line of research, we must provide the pioneers in this field with systematic information on how different entrepreneurs behave differently to produce different outcomes. Only then can they begin to simulate how an economy behaves in the aggregate when individual entrepreneurs are heterogeneous.

Second, their work will gain increasing importance as new challenges (e.g., climate change) and shocks (e.g., COVID-19) hit our economies. Macro-economics will be pushed towards including entrepreneurs in its models if it wants to speak to the questions that these real-world challenges pose. It is entrepreneurs who routinely handle (deep) uncertainty, creatively destroy obsolete technologies and organizations and channel society’s resources towards new value creation. This process requires creativity and resources for experimentation, while a lack of (new) knowledge and ideas is typically not what is holding them back. The bottleneck in innovation is not ideas but entrepreneurship (Schumpeter, 1911). Moreover, it is the same people, traits, and activities helping an economy first resist and later recover from unanticipated shocks that will also help it transition to a new paradigm (Korber and McNaughton, 2017; Hartmann et al., 2022).

Studying the behavior of the aggregate economy at the local, regional, national, or global level will increasingly demand an understanding of the processes of transition and adaptation that both, in essence, boil down to processes of rapid change under deep uncertainty. Institutions and the deep historical roots of long-run development processes become much less relevant in that context. Entrepreneurship may be a proximate cause of long-run economic development, but it is not a proximate cause of adaptation and change.

#### … if we adopt a behavioral definition of entrepreneurship

To support this development in macro-economics, however, entrepreneurship scholars need to adopt an unambiguous and exogenous definition of entrepreneurial behavior (Sanders, 2022). The emphasis on empirically convenient but theoretically fuzzy definitions of entrepreneurship is what holds its impact on macro-economics back. Macro-economists need to know what it is that entrepreneurship does to model its aggregate impact. Therefore, we need to be explicit and unambiguous on what it is that entrepreneurs *do*, that has macro-economic implications. To the extent that they are macro-economically relevant, entrepreneurs are agents of change (Schumpeter, 1911). And to play that role, they must challenge the status quo. They may be successful or fail in doing so, but that behavior defines an entrepreneur in a macro-economically relevant sense. Only then can we show convincingly that it is the absence/presence of such entrepreneurial behavior that explains how different economies respond differently to similar shocks. For a macro-economist, it will not do to define entrepreneurs as people starting, owning, or managing a (small and/or young) firm or being self-employed. That status can be achieved regardless of the actions taken. It is also unproductive to “measure” entrepreneurship as a complex index of many variables that we find or select to positively correlate with long-run development, growth, and/or innovation in the aggregate. Defining entrepreneurship by its outcomes ignores the trial and error involved and creates an endogeneity issue from the outset. “Entrepreneurship is what entrepreneurship does,” Forrest Gump’s mama would say. So, let us define entrepreneurs by their (macro-economically relevant) actions. If you challenge the status quo (successfully or not) you are an entrepreneur. If you do not, you are not.

### Entrepreneurship and public policy

#### From the Second World War to Birch (1981)

Policy had little reason to prioritize entrepreneurship in the first decades after the Second World War. The economic world was obsessed with scale economies: bigger firms were more productive than smaller ones. A destroyed world needed a fast and reliable recovery, and economies of scale were an easy way to deliver it. The great lesson that ultimately led the Allies to victory was that scale economies prevail. Victory went to those able to mass produce warships, tanks and aircraft in the least amount of time with the most efficient use of resources. Coming out of a devastating Second World War, the gulf between the communist East and capitalist West ushered in a new world order. However, one thing both sides agreed upon, bigger was better, and when it came to bigger, the communist part of the world showed the capitalist part of the world all of the advantages of large-scale production (Thurik et al., 2013). This was the golden age of the so-called “managed economy”: economic development and growth depended upon big firms, with the government there to protect them and to not hurt their employees too much (Audretsch and Thurik, 2001).

The terms “young firms” and “starting firms” were barely used. Even the word “entrepreneurship” was virtually non-existent. Small firms were useful to stack away a substantial proportion of employment, to play a role in the public safety of city centers and to signal to large firms what consumers preferred. The role of small firms was seen as social rather than economic (Thurik, 2009). This was evidenced by the passage of the United Small Business Act in 1952, which created the United States Small Business Administration to preserve and protect what was considered to be an endangered species of business. Small and new business was valued more for its contributions to a democratic society than for efficiency, productivity, innovation, and growth (Audretsch and Moog, 2022).

In this world involved with recovery and reconstruction from the Second World War, rebuilding infrastructure, focusing on heavy manufacturing and engaging labor in repetitive, standardized tasks fuelled unprecedented rapid growth that had not been seen for decades. However, this antidote stopped working as a more nuanced view that social and economic values encompass a broader spectrum of goals in the 1960s, such as distribution and “social makeability.” The pop culture, hippy movement, flower power, and student revolts such as those in Paris in 1968 ushered in a new urgency for societal change. However, a transition to what? In addition, transition to a newer economy was needed because on the horizon loomed the economic crisis in the 1970s, which jolted the OECD countries out of the post-war equilibrium. Unemployment reached double digits for the first time since the Second World War. Both scholars and policy-makers looked to the tried and true stalwart, dominant large corporations, to restore the prosperity and jobs that were proving to be so elusive. A stagnant policy thinking reflected a similarly stagnant economy. The scientific metabolism of those who study competitiveness and growth had come to a halt.

In 1981, Birch startled this phlegmatic policy world by identifying new and small businesses as the primary source of new jobs (Birch, 1981). Dismay with this new finding was mixed with the first signs of relief that perhaps a new stalwart was on the horizon. However, jobs are not sufficient to create competitiveness. In *Made in America: Regaining the Productive*, the MIT Commission on Productivity argued that restoring the competitiveness of the industrial giants in the manufacturing industries that had carried post-war prosperity was the key to reigniting economic prosperity (Dertouzos et al., 1989).

#### From small firms to ambitious entrepreneurship

Therefore, while public policy thinking in the 1980s was moving away from the 1960s’ fixation on the dominant role of large firms and the 1970s’ fixation on the role of government distributing and supporting “social makeability,” it still offered few clues of what economic dynamics really is and how it can enhance competitiveness. Entrepreneurship was scarcely discussed and certainly not prioritized. The question of why so many small businesses still existed should have been the central research question of the time. Businesses do not just exist for social reasons as justifications for public policy. Businesses do not just exist because their owners are too stubborn to exit. The essential contribution of small business and entrepreneurship went largely unnoticed—the 1980s produced no lasting influencers.

A decade later, as the competitiveness of firms and nations was challenged by the advent of globalization (Porter, 1990), both scholars and thought leaders in business and public policy turned to innovation as the catalyst for restoring economic viability. Once again, research uncovered small and new firms as making an unexpected but robust contribution to the coveted economic goal, innovative activity (Acs and Audretsch, 1988; Acs and Audretsch, 1990).

The discovery that small firms played a role in the creation of jobs and later in the creation of newness completely overturned policy thinking. Of course, big firms create newness in their sophisticated laboratories, but it does not always work like that. Swarms of small firms experiment, learn from each other, die or not, grow or not, merge or not, and, in the end, sometimes something new happens. This newness may be swallowed up by some large firm that continues the road to the market (Audretsch, 2007). We then talk of an innovation. With this new understanding of the contribution to innovation and competitiveness contributed by small and new firms, a concomitant shift in public policy occurred, prioritizing and starting and nurturing new firms, encouraging, in particular, their aggregation in industrial zones and business parks.

In retrospect, it is remarkable how smoothly this discovery of the economic value of smallness was absorbed by the public and by policy-makers. It was never regarded as a delusion or a mirage, or worse, a conspiracy theory “avant la lettre.” In the 1980s, the supply of studies on entrepreneurship and small business economics exploded. By the 1990s, a marked demand for small business studies had developed. At the turn of the century, demand had caught up to supply, a life changer for academic entrepreneurship and small business researchers.

The first decade of the current century witnessed a less spectacular change in thinking. What are small firms? Big firms that are just small? Of course not. A small firm is built around a person, whereas its larger counterpart has many stakeholders. The concept of the entrepreneur was reinvented, and with it, the entrepreneurial economy (Audretsch and Thurik, 2000). Just as Taylorism (Taylor, 1911) had removed the need for agency and autonomous decision-making in ushering in the field of management nearly a century earlier, the burgeoning field of entrepreneurship instead celebrated a focus on the humanness of individuals and people. Public policy followed by shifting to target persons rather than firms.

The consequences of this shift in policy orientation were gigantic. Large firms were no longer the focal point of the economy, society, and policy initiatives. The entrepreneur became the persona causa (again) of economic prosperity. Business schools, for instance, had to reorient from large firms towards single persons. This took immense energy through internal conflict. Ultimately, they were successful. All leading business schools now house eminent teams of entrepreneurship scholars. A by-product of this double switch—from large incumbents to small young firms and from firms to persons—was the establishment of incubation centers. These centers have been at the forefront of a multitude of policy initiatives for the better part of the current century.

In the second decade of the current century, sentiment changed yet again. Entrepreneurship alone may be relevant, modern and heroic, but it is not enough to sustain growth in developed economies. Thus, the focus shifted to ambitious entrepreneurship: innovating, exporting, growing by using clever forms of cooperation, flexibilization, and the exploitation of modern information and communication techniques (Stam et al., 2011; Hermans et al., 2015). Moreover, recognition grew that entrepreneurship is not the sole privilege of small young firms. It can also play a role within large incumbent firms and within not-for-profit organizations. It plays a role everywhere.

#### Deep tech and the entrepreneurial society

Looking at the future, the question is what catch word can be connected to entrepreneurship in the third decade of this century?

The second information and communication technology revolution—which basically made the technology portable, miniature, cheap, and user friendly—was not masterminded. Herds of small firms housing nerds and unrelenting entrepreneurial energy played a crucial role. The message from a world where hundreds of millions live at—or even below—sea level can only be this: we need climate-tech businesses in the hope that they create “iPhone”-like or rather “Netscape”-like moments (The Economist, 2021). Falling out of the sky in the mid-1990s, the Netscape web browser was a revolution that connected people to whatever they wanted. For the next big moment, we need invention and innovation to explode. In addition, for that to occur, we need herds of nerds surrounded by entrepreneurial vibrancy. Otherwise, the decarbonizing transition will not happen.

Large firms have considerable cash, vision, “Ausdauer,” and brains at their disposal. Will they contribute to decarbonizing with their commitments to invest time and pledges to invest money in “greeneries this, footprints that, responsibilities this, and net-zero that”? Their embedment in current technology may not be compatible with radical thinking. The herds of nerds showered by risk-tolerant venture capital and nurtured by public emotions of urgency may be a better route to creative destruction. Few people have an idea about the technologies of and behavior around decarbonizing. If we did have any idea, we would not need the herds of nerds doing crazy things.

There surely is more capital looking for brains than brains looking for capital. The world of capital fully understands that spreading risks by loaning to many technologically diverse firms is a better option than going with the understandable and recognizable flow and loaning to technologically similar firms. More importantly, it has been shown many times that the fruits of creative destruction can generate formidable profits.

Again, what is the catch word of the present decade? To entrepreneurship scholars, every decade can be connected to entrepreneurship in some way. With the grand challenges of climate change ahead of us, including the need for decarbonization and the probable consequence of increasing societal inequality, impact-driven entrepreneurship and innovation are needed more than ever. Deep technology will play an important role in this regard. Hence, we use the term “impact-driven deep tech entrepreneurship,” which points to entrepreneurial activities of a scientific, technical nature aimed at products—and hence markets—that do not yet exist and that not only create economic value but, even more importantly, have a strong impact on society with its many grand challenges.

This view on the crucial role of entrepreneurship in humankind’s survival leaves no room for the view that entrepreneurial society is over (Naude, 2022), that the domination of megafirms will surely increase, that entrepreneurial energy will fade in a world where the lowest caste consists of jobbers and freelancers, and that large government is returning. In other words, this view conflicts with those who maintain that Western democratic liberalism has become sclerotic (Audretsch and Moog, 2022).

Despite many attacks from both the right and the left, Western democratic liberalism is still in full swing, as is entrepreneurial society. We will need it to unleash “impact-driven deep tech entrepreneurship.” In other words, and in the terminology of democratic liberalism itself, we need to get out of the way while “impact-driven deep tech entrepreneurship” unleashes itself. Its much-needed successes will provide the oxygen for the next phase of Western democratic liberalism.

Table 1 shows 8 decades of entrepreneurship in public policy since the Second World War in terms of “catch” words.

**Table 1:** Entrepreneurship-related “catch” words in public policy since World War II

1950s	Reconstruction and growth
1960s	There is more than growth
1970s	Distribution and “makeability”
1980s	Small firms
1990s	New and young firms
2000s	Entrepreneurship
2010s	Ambitious entrepreneurship
2020s	Impact-driven deep tech entrepreneurship

#### Rising to the challenge

The policy priority has evolved from ignoring entrepreneurship to recognizing its role, from jobs to subsequently innovation, competitiveness, and growth, from firms to persons, from persons to ambitious persons and now, it can be hoped, from activities concerning existing to not yet existing markets. This change has been in sync with a growing awareness that entrepreneurship contributes to sustainability and the inclusion of socially excluded demographic groups and, most recently, a free and democratic society (Audretsch and Moog, 2022). The research community not only responded with a robust body of literature linking the most compelling issues of the “Zeitalter” to entrepreneurship, it often led the way to help society to adapt its awareness and policy to refocus. In other words, the research community was continuously linked to new policy goals and contributed to defining and evaluating them and influencing the “Zeitgeist.” Thus, entrepreneurship has emerged as a priority for public policy in rising to the challenge of a myriad of broad social and economic challenges.

This evolution of entrepreneurship policy can be viewed through the lens of equilibrating the overall policy approach with the underlying economic forces and challenges characterizing each period. In the 1950s, this involved recovery and reconstruction from the Second World War, with an emphasis on infrastructure, heavy manufacturing and rapid growth. The priority of high growth gave way to a more nuanced view that economic and social values encompass more than just growth in the 1960s, which in turn transitioned into an emphasis on distribution and “makeability” in the 1970s. Entrepreneurship was scarcely discussed and certainly not prioritized over this time span of more than 3 decades. However, by the 1980s, the discovery that small firms are drivers of job creation thrust them into a focal point of policy. As the driving force of economic growth shifted towards innovation in response to the onset of globalization in the 1990s, the policy priority again shifted to new and young firms. Thus, by the turn of the century, the contemporary policy focus on entrepreneurship emerged, which has more recently become refined by prioritizing ambitious entrepreneurship and “deep tech” entrepreneurship.

Thus, by staying relevant to society and providing policy solutions, entrepreneurship research has evolved from being a largely tangential and marginal field of inquiry to one of the most dynamic and robust fields in the social sciences, policy and management. The influential entrepreneurial ecosystem literature is one of the best examples of the crucial role of entrepreneurship in policy thinking both from a policy and scholarly perspective (Wurth et al., 2022). Without a strong and compelling body of literature, the mandate for public policy to ignite entrepreneurship to enable society to rise to the challenges posed by some of the most daunting and gripping problems would not have materialized. At the same time, it has been the ability of the scholarly field of entrepreneurship to address the most pressing societal problems that has no doubt fuelled its emergence as one of the most important fields of inquiry in management and the social sciences. This two-way traffic is sometimes documented but only implicitly so. See for instance Terjesen et al. (2016).

## Synthesis of how entrepreneurship research impacts other fields

The goal of this article is to showcase how modern entrepreneurship research contributes to the development of other academic fields. This was achieved based on observations from a carefully selected group of interdisciplinary entrepreneurship scholars with a strong grounding in fields outside the core of entrepreneurship research. Based on these contributions, we identified five areas and ways in which entrepreneurship research had and still has an impact on the development of other disciplines.

Entrepreneurship research contributes to the development of other fields by *providing new ideas for the consequences of phenomena* that are already well studied in these fields. As explained in Section 2.1, ADHD has been studied in clinical psychology for decades. Entrepreneurship research has taken a different perspective and looked at how ADHD can actually be beneficial for starting an (entrepreneurial) career. This is a new perspective, as clinical psychology has traditionally only looked at the negative occupational aspects of ADHD. Entrepreneurship research has provided a fresh perspective on clinical psychology research on ADHD and spurred new empirical research with a whole range of new outcome variables ranging from entrepreneurship intention (Verheul et al., 2015) to entrepreneurial orientation (Wismans et al., 2020) and entrepreneurial performance or outcomes (Wiklund et al., 2016; Patel et al., 2021). A similar conclusion can be made about the relationship between entrepreneurship and occupational health research, as explained in Section 2.2. Entrepreneurship as an “extreme” work setting can enhance both ill- and well-being, which introduces a new trade-off that has thus far not been a focus of occupational health research. Again, entrepreneurship research inspired a discipline far away from entrepreneurship research to investigate a consequence that was not the focus of occupational health research. Future clinical psychology and occupational health research can build on these ideas and apply them to other phenomena studied in their respective disciplines. For example, occupational health research could analyze the ill- and well-being trade-off that exists with extreme sports athletes, artists, and managers of large firms. Clinical psychology research could study the relationship between entrepreneurship and dyslexia, autism, and narcissism (e.g., Leung et al., 2021). Similar situations or opportunities also exist with other fields. For example, family or educational sociology could analyze the impact of familial (Aldrich and Cliff, 2003) or educational socialization on entrepreneurship-related outcome variables. Another recent example is existentialism: entrepreneurs tend to view their business as the center of their existence. Three concepts have been introduced to show the link between existentialism and mental and physical health: subordination, suffering, and “salutogenesis” (Torrès et al., 2022). The eco-systems literature developed a focus on the role of entrepreneurship (Wurth et al., 2022) with policy and scholarly perspectives. A last example is the field of spirituality and religion that has looked at how religion and spiritual beliefs influence entrepreneurship processes and outcomes. For a summary, see Block et al. (2020).

Context has emerged as being crucial for the scholarly field of entrepreneurship. An important finding in the present study is that for other research fields, entrepreneurship is the context. Entrepreneurship is for other fields an interesting, *unique and extreme context* in which to analyze relationships that are already analyzed in a “normal” context. For example, research on the relationship between work demands and well-being can benefit from using an entrepreneurship context. With innovative entrepreneurship, for example, uncertainty is often particularly high, and evidence about the relationship between entrepreneurial work demands and entrepreneurial well-being can help to explain how work demands impact well-being in a setting of high uncertainty. In this way, entrepreneurship research contributes to the core literature on occupational health. A similar argument exists with genetic epidemiology (see Section 2.3), which is concerned with how particular combinations of genes influence individual behavior. Entrepreneurship, which is associated with high uncertainty and risk, can be used as a unique context to analyze how genetic dispositions impact uncertainty tolerance or risk aversion. Alternatively, entrepreneurship can be used as a contextual moderator explaining the relationship between two variables of interest outside the scope of entrepreneurship research. For example, educational science might be interested in learning about the effect of education on earnings or job satisfaction. Using innovative entrepreneurship as a unique and extreme context may help to explain how education impacts earnings or job satisfaction in a dynamic environment characterized by high environmental uncertainty.

Entrepreneurship can also be an *antecedent that explains phenomena of interest* in other fields. For example, as illustrated above in Section 2.4, entrepreneurship—in particular high-impact successful entrepreneurship—has the power to shape societal culture, leading to an entrepreneurial society. A good example would be to analyze how Silicon Valley entrepreneurship and/or the German Mittelstand entrepreneurship model (Pahnke and Welter, 2019) shape societal values about individualism and power distance. A related example would be to analyze how entrepreneurship impacts societal or regional inequality (Halvarrsson et al., 2018; Lippman et al., 2005), which is at the core of disciplines such as sociology and political science. This argument holds not only for the soft sciences but also for the hard sciences. If one wants to understand why certain technologies diffuse in the market and become technological standards and others do not, entrepreneurship research provides a fresh perspective. Entrepreneurs can be change agents introducing new technologies and innovations into the market, impacting technology diffusion and competition (Block et al., 2017; Block et al. in press; Miller and Garnsey, 2000; Schumpeter, 1911). In this way, entrepreneurship research contributes to technology research but also to an understanding of industrial structures, which is at the core of competition and industrial organization research (see Section 2.5).

Sometimes, entrepreneurship research *challenges the fundamental assumptions of other disciplines,* contributing to a more realistic and empirically grounded micro-level underpinning of theoretical models. As described above in Sections 2.5 and 2.6, the empirical findings from entrepreneurship research can be seen as a game changer of industrial organization and macro-economic theory, which have for years largely ignored the role of the entrepreneurs managing young and innovative firms that bring innovation to the market and destroy existing market equilibria. A similar comment can be made about (corporate) finance research, which is influenced by theorems such as the Modigliani-Miller theorem (Modigliani and Miller, 1958) stating that the market value of a company does not depend on its capital structure and that it does not matter whether a firm finances its growth through debt or equity or through internal or external sources of finance. Entrepreneurship research has shown that this assumption and statement does not hold for innovative, high-growth, and high-impact firms facing high levels of uncertainty. The result is the emergence of entrepreneurial finance as an important and fast-growing research area at the intersection of entrepreneurship and finance research, investigating how innovative firms use venture capital, business angel money, crowdfunding, initial coin offering and other entrepreneurial financing players and tools to finance innovation and growth (Block et al., 2018). The case of crowdfunding research clearly illustrates the important role of entrepreneurship research as a catalyst for finance research on crowdfunding, as many of the early influential papers on crowdfunding appeared in the leading entrepreneurship, and not finance, journals (e.g., Belleflamme et al., 2014; Mollick, 2014). The example of entrepreneurial finance, however, also illustrates that, sometimes, parallel universes can exist where two disciplines analyze the same set of questions while largely ignoring developments in the other discipline.

Entrepreneurship research provides other disciplines with the opportunity to show that their research matters from a practical perspective. As described above, it changed the perspective of policy research explaining what it takes to build an entrepreneurial society and economy. Policy-makers around the world were fast to jump on this train and grab the terminology of entrepreneurship (research), such as start(ing)-up, scale-up, entrepreneurial society, (corporate) venturing, or seizing opportunities. By analyzing entrepreneurship variables as outcome variables, neighboring disciplines took the chance to gain *legitimacy in the practical world* of business and policy decision-makers and research funders. There is hardly any contemporary research funding agency that does not emphasize the third mission of universities: knowledge transfer and the practical impact of the funded research. Entrepreneurship through new ventures or existing organizations has become the prevalent mode for the commercialization of university research and knowledge. Relatedly, scholarly societies such as the Strategic Management Society took the chance to create journals such as the *Strategic Entrepreneurship Journal* at the interface of their own and the entrepreneurship field. It remains to be seen what this means for the core leading entrepreneurship journals. Thus far, it seems that these journals benefit from this development given the steady rise of their impact factors, which are now even higher than those of some of the established and general management and economics journals.

Table 2 below summarizes the above discussion and provides an overview of how entrepreneurship research has an impact on other fields.

**Table 2:** Impact of **entrepreneurship research** on other **academic fields**

**What entrepreneurship research contributes to other academic fields**	**How this impacts the development of other academic fields**
Provision of new ideas about consequences of well-studied phenomena	New and important, dependent, moderating and mediating variables
An interesting, unique and extreme contextual situation in which to analyze well-studied research questions and models	New and deeper insights about the strength of relationships in well-established research models
An important context that can explain the emergence of phenomena	New and deeper insights about the emergence of *new* phenomena
Challenging the fundamental assumptions of well-established theoretical models	A better, more robust grounding of fundamental theoretical assumptions, ultimately leading to more realistic theoretical models with higher predictive power
A change of the mandate, tool-box, and terminology	More legitimacy in the *practical* world through new goals and questions of high practical relevance justifying the research impact

What explains the success of entrepreneurship research having an impact on other fields? What are the mechanisms behind the two-way traffic? It is hard to give a definite answer but it surely helped that many of the early entrepreneurship research scholars had a disciplinary grounding *outside* the field of entrepreneurship research which facilitated the communication and led to many entrepreneurship publications outside the core entrepreneurship journals.^4^ It also resulted in joint conferences, workshops, and entrepreneurship sessions in conferences of other disciplines giving visibility for entrepreneurship research^5^ and leading to multidisciplinary research teams.^6^

## Conclusion and outlook

Our article shows that entrepreneurship research indeed influences other academic fields. It questions core assumptions of other fields and provides new insights about the antecedents, mechanisms, and consequences of important phenomena. Moreover, entrepreneurship research helps legitimize other fields in the practical world.

Given that entrepreneurship research seems to impact other fields, what does this mean for the field of entrepreneurship research itself? *First*, it underscores the need for (theoretical and empirical) sophistication as the impact of entrepreneurship research goes beyond its own discipline. Yet, too much sophistication may be “too-much-of-a-good-thing” and reduce the impact of entrepreneurship research on other fields and (even more) on public policy. There is a fine line between scientific sophistication and relevance of entrepreneurship research for other fields and policy-makers. *Second*, given the ability of entrepreneurship research to establish a two-way traffic between fields, a danger looms of losing its own identity and becoming unfocused and eclectic. Entrepreneurship research should avoid this and keep its own theoretical and empirical concepts, measurements, and grounding.^7^*Third*, the two-way traffic also has implications for the evaluation of the quality of entrepreneurship research and its makers. It is not enough to measure only the scholarly impact in entrepreneurship research or the broader fields of management and economics. Our study shows that the entire social sciences could be included as well as some fields from the hard sciences.

How will the relationship between entrepreneurship and other fields evolve? The spectrum of possibilities seems endless. It ranges from entrepreneurship research becoming so mainstream and ubiquitous that it loses its identity and meaning, eventually disappearing, to entrepreneurship research further developing its own identity and agenda constantly bringing its methods, tools and questions into other fields, inspiring them to come up with new answers to the important questions in these fields. Surely, the future will tell us which direction entrepreneurship research as a discipline will take. A host of new challenges, including environmental sustainability, social inclusion and income equality, confront a bewildered society. If the past is indeed prelude to the future, we can look to the promise of entrepreneurship research to continue its influence in shaping thinking and ideas across a broad spectrum of academic disciplines and fields.

## Footnotes

1 We took all journals in the management- and business-related categories from the SSCI and ranked them by the 2021 impact factor (IF). The respective journals listed under the top 100 are *Journal of Business Venturing* (IF: 13.12), *Entrepreneurship Theory and Practice* (IF: 9.99), *Small Business Economics* (IF: 7.10), *Journal of Small Business Management* (IF: 6.88), International Small Business Journal (IF: 6.41), Entrepreneurship and Regional Development (IF: 6.41), International Journal of Entrepreneurial Behavior and Research (IF: 6.30), International Entrepreneurship and Management Journal (IF: 6.15), and Strategic Entrepreneurship Journal (IF: 5.76).

2 Cases could clearly be made for economic geography, sociology and health in the sense that these fields may have benefitted from the entrepreneurship view. Geography journals such as Regional Studies, Economic Geography, Journal of Economic Geography and European Planning Studies report many entrepreneurship articles. Already in 2008, the interactions between sociology and entrepreneurship have been dealt with by Sorensen and Stuart (2008).

3 The present paper focuses on research, but clearly the “academic field of entrepreneurship” is bigger than that. It also plays a role in areas such as teaching and the institutional infrastructure from incubation centers to entire eco-systems. For instance, entrepreneurship research has had an impact on educational methods such as challenge-based learning and a competence-based approach to teaching which again influenced research on entrepreneurship education. The two-way traffic between entrepreneurship and the eco-systems literature is an even better example of links not just research based but also having solid policy underpinnings. See the transdisciplinary entrepreneurial ecosystem research program proposed in Wurth et al. (2022).

4 Zoltan Acs and David Audretsch moved over from the field of industrial economics, Roy Thurik from marketing, David Story from geography, just to name a few.

5 Experimental entrepreneurship sessions during the American Economic Association meetings, the European Association for Research in Industrial Economics meetings and the Dutch Ecozoek meetings of the late 1980s come to mind as examples of how entrepreneurship discovered itself as some subfield within platforms aimed at a broader field.

6 Publications such as Van der Loos et al. (2013) and Obschonka et al. (2015) are examples of multidisciplinary teams aiming to connect entrepreneurship to genetics and culture, respectively.

7 Also, here there is fine line between the identity of entrepreneurship and the ability to connect to other disciplines. The relevance to other disciplines is sometimes hampered by ambiguous definitions of entrepreneurship, business owners, self-employment, ecosystems or whatever. The use of multiple proxies for a theoretical concept is permitted of course but only if they are clearly defined. See also the section on macro-economics above.
